# Knowledge and Perceptions of Psychiatric Disorders: Influence on the Demand for Care

**DOI:** 10.1192/j.eurpsy.2026.11829

**Published:** 2026-07-31

**Authors:** L. Azizi, O. Seyar, Z. Bencharfa, F. Omari

**Affiliations:** Hôpital Arrazi, Salé, Morocco

## Abstract

**Introduction:**

Stigma associated with mental disorders remains a major public health barrier. It manifests as negative attitudes, prejudices, and discrimination that limit access to psychiatric care. Despite progress in the understanding and treatment of mental illness, many people avoid seeking help for fear of being judged, marginalized, or labeled. This situation contributes to delayed diagnosis, worsening of symptoms, and a decrease in patients’ quality of life.

**Objectives:**

- To assess how stigma influences individuals’ decisions to consult a psychiatrist.
- To identify social, cultural, and economic factors that reinforce or mitigate stigma.
- To analyze the consequences of delayed or absent treatment on patients’ clinical outcomes.
- To propose interventions to reduce stigma and improve access to psychiatric care.

**Methods:**

To meet these objectives, we conducted a **descriptive study** that collected **102 cases** at the Arrazi Psychiatric Hospital in Salé.

A structured data collection sheet was used.

**Results:**

**Sociodemographic characteristics:** The majority of patients were female (66.3%). The average age in our population was between 20 and 30 years (62.4%), with a university education level (79.2%). A total of 56.5% were employed.

**Perception of stigma:** In our study, 65.3% of respondents believed that people with psychiatric disorders are stigmatized in society. 5.9% did not think these individuals are stigmatized, while 28.8% were unsure. In addition, 67.4% had witnessed situations where a person with a psychiatric disorder was treated unfairly. 20.8% had not witnessed such unfair treatment, while 11.8% were uncertain.

**Access to psychiatric care:**

- 28.8% experienced feelings of isolation within the family.
- 26.6% felt shame or embarrassment.
- 22.2% feared judgment from society.
- 20% reported a lack of financial resources.
- 2.2% experienced feelings of isolation within the family (duplicate response in the data).

**Impact of stigma on quality of life:**

- 30.1% were affected by a reduction in self-esteem and confidence.
- 27.02% reported increased stress and anxiety.
- 22.97% experienced impaired social relationships.
- 19.36% faced limited employment or educational opportunities.
- 0.45% reported worsening of their illness, which could have harmful consequences.

**Conclusions:**

Stigma is a major barrier to accessing psychiatric care. It generates a vicious cycle: delayed treatment, clinical worsening, social isolation, and persistence of prejudice. Combating stigma requires a comprehensive approach that combines awareness campaigns, training of healthcare professionals, psychoeducation programs, and community-based actions. Reducing stigma is not only an issue of individual mental health but also an imperative for social justice and public health.

**Disclosure of Interest:**

None Declared

